# Single and double layer centrifugation improve the quality of cryopreserved bovine sperm from poor quality ejaculates

**DOI:** 10.1186/s40104-016-0088-6

**Published:** 2016-05-05

**Authors:** Alessia Gloria, Augusto Carluccio, Laura Wegher, Domenico Robbe, Giovanni Befacchia, Alberto Contri

**Affiliations:** Faculty of Veterinary Medicine, University of Teramo, Località Piano D’Accio, 64100 Teramo, Italy; Provincial Breeders Federation of Trento, Via delle Bettine 40, 38121 Trento, Italy; Associazione Regionale Allevatori d’Abruzzo (ARA), S.S. 17 Est loc., Onna, L’Aquila, Italy

**Keywords:** Bull, Cryopreservation, Density gradient centrifugation, Iodixanol, Semen analysis

## Abstract

**Background:**

Density gradient centrifugation was reported as a technique of semen preparation in assisted reproductive techniques in humans and animals. This technique was found to be efficient in improving semen quality after harmful techniques such as cryopreservation. Recently a modified technique, single layer centrifugation, was proposed as a technique providing a large amount of high quality spermatozoa, and this treatment was performed before conservation. Single layer centrifugation has been studied prevalently in stallions and in boars, but limited data were available for bulls. Occasionally bulls are known to experience a transient reduction in semen quality, thus techniques that allow improvement in semen quality could be applied in this context. The aim of this study was the evaluation of single layer and double layer centrifugation by the use of iodixanol, compared with conventional centrifugation and non-centrifuged semen, on the sperm characteristics during the cryopreservation process in bulls with normal and poor semen quality.

**Results:**

Single layer centrifugation and double layer centrifugation both significantly increased the percentage of normal spermatozoa and decreased the percentage of non-sperm cells in poor quality samples, while both were ineffective in those of normal quality. Sperm characteristics in poor quality samples increased after single layer centrifugation and double layer centrifugation, reaching values similar to those recorded in normal samples, and this trend is maintained after equilibration and after cryopreservation. On the other hand, SLC and DLC resulted in a consistent reduction in the spermatozoa recovered, and this resulted in a reduction of the absolute amount of spermatozoa cryopreserved in the normal samples, without a clear improvement in sperm characteristics in this type of sample.

**Conclusions:**

These data suggested that both SLC and DLC could be performed in practice, but their application should be limited to the cases in which the quality of the spermatozoa recovered is more important than the total amount of spermatozoa.

## Background

Artificial insemination (AI) is generally considered to be the technique that has contributed the most to the improvement of animal reproduction. This technique can result in pregnancies only if the insemination dose contains sufficient viable spermatozoa capable of reaching the site of fertilization [1]. In this context, semen analysis is the most commonly used procedure to evaluate male fertility potential in humans and animals [2–5].

Density gradient centrifugation through colloids is one of the procedures recommended by the World Health Organization for the preparation of human spermatozoa used in assisted reproduction [6]. In animals, this method has been suggested as a potential means of improving the quality of sperm after cryopreservation [7–9]. Recently, a modified gradient centrifugation technique, single layer centrifugation (SLC), was proposed to improve the semen quality before conservation and AI [1, 10]. This technique provides high quality sperm, with an increased number of motile and membrane-intact spermatozoa. On the other hand, a significant loss of spermatozoa during the centrifugation procedure was reported [1, 11], and the absolute number of efficient sperm recovered after this procedure was lower than in conventional centrifugation [12]. This procedure should therefore be reserved for cases in which the sperm characteristics after density centrifugation are more important than the total amount of spermatozoa recovered. Evidence has suggested that the recovery rate of spermatozoa after centrifugation is related to the g-force applied. On the other hand, an excessive centrifugation force reduced the viability of spermatozoa, probably because of the physical pressure against the wall of the tube [13–15].

In a study performed on bulls, SLC was found to increase sperm mitochondrial membrane potential, reactive oxygen species (ROS) production, and protein phosphorylation, and to decrease sperm chromatin damage, while other parameters such as sperm motility, membrane integrity and morphology remained similar [16]. However, these effects were recorded soon after centrifugation, but no data on the long term effects or the impact of this procedure on cryopreservation were verified.

The primary aim of this study was the evaluation of the effect of SLC and DLC on morphological and functional quality of fresh spermatozoa in bulls with normal and poor semen quality. Secondly, the effect of centrifugation (SLC, DLC, and conventional centrifugation) on bovine semen characteristics after equilibration and cryopreservation was evaluated.

## Methods

### Animals and preliminary semen evaluation

This study was performed on 20 Swiss Brown bulls belonging to Superbrown Consorzium Bz/Tn (1.5 to 5 yr old). The bulls were housed in the Alpenseme AI Center of the Provincial Breeders Federation of Trento (Ton, Trento, Italy). Animals used in this study were managed according to the National Law for Animal Welfare and Protection (Italy).

Semen was collected using an artificial vagina and evaluated. The volume was measured from the graded collection tube soon after collection; concentration was determined using Accucell photometer (IMV Technologies, L’Aigle, France) after dilution 1:100 with saline solution. Progressive motility (PM) was evaluated subjectively (magnification: × 400) at 37 °C using phase contrast microscopy, after dilution with pre-warmed Bioxcell (IMV Technologies) at 80 × 10^6^ sperm/mL. Morphology was evaluated after dilution at 80 × 10^6^ sperm/mL with 0.9 % NaCl solution and 3 % glutharaldehyde using phase contrast microscopy (magnification: × 1,000) [17, 18].

Bulls were classified with normal semen quality (N), with subjective progressive motility ≥ 70 % and normal sperm morphology ≥ 70 %, or poor semen quality (P), with PM and morphology lower than the thresholds. Part of the ejaculate for each bull was used for the study.

### Centrifugation procedures

To evaluate the effect of SLC and DLC on sperm characteristics after centrifugation, ejaculates were divided into four aliquots and three of them were diluted with a modified Tyrode’s medium (TALP) [19] at 30 × 10^6^ sperm/mL. The fourth (Control non-centrifuged - NC) sample was diluted at 30 × 10^6^ sperm/mL with Bioxcell (IMV Technologies) and was processed without centrifugation.

The Optiprep (Axis-shield, Olso, Norway), as iodixanol 60 %, was diluted with TALP to reach a concentration of 15 % iodixanol (top layer - 1.075 g/mL, pH 7.4, 302 mOsm) and of 35 % iodixanol (bottom layer – 1.192 g/mL, pH 7.3, 298 mOsm).

For SLC, 4 mL extended semen were layered over 4 mL of iodixanol 15 % in a 15 mL Falcon tube, taking care not to allow the semen to mix with the colloid. After centrifugation at 1,000 × g for 20 mins, the supernatant (seminal plasma plus extender) and most of the colloid were discarded. The sperm pellet was resuspended at the original volume with Bioxcell (IMV Technologies).

For DLC, 4 mL of iodixanol 15 % was placed first, then 4 mL of iodixanol 35 % was gently deposited at the bottom of the tube; finally, 4 mL of extended semen were layered on the top of the iodixanol solutions. After centrifugation at 1,000 × g for 20 mins, supernatant and the bottom 35 % of iodixanol were removed and sperm pellet was resuspended at the original volume with Bioxcell (IMV Technologies).

Conventional centrifugation (CC) was performed by the centrifugation of a 4 mL aliquot of diluted semen at 600 × g for 10 mins without colloid, and then resuspended at the original volume with Bioxcell CSS I and II (IMV Technologies).

After processing, sperm characteristics were evaluated in all samples.

### Cryopreservation

All four samples from each bull were transferred to a beaker containing water at room temperature, then were cooled passively to 5 °C for 1 h. Samples were equilibrated at the same temperature for an additional 2 h. At the end of the equilibration period, one aliquot from each sample was evaluated. The equilibrated samples were loaded in 0.25 mL French straws and frozen in a programmable nitrogen vapor freezer (DigitCool, IMV Technologies), using a conventional freezing rate [20]. The straws were then plugged into liquid nitrogen and stored for at least 7 d. Ten straws for each treatment were thawed at 37 °C for 60s in a waterbath, incubated for a further 10 mins at 37 °C and then evaluated.

### Semen evaluation

The evaluation of the sperm kinetics, morphology, viability/acrosome integrity and mitochondrial membrane potential were performed on samples after centrifugation, equilibration, and cryopreseration.

### Recovery rate

The recovery rate was calculated, measuring sperm concentration by a Burker counting chamber before and after the centrifugation, as follows:

$$ \left(\mathrm{concentration}\ \mathrm{after}\ \mathrm{centrifugation}/\mathrm{concentration}\ \mathrm{before}\ \mathrm{centrifugation}\right)*100. $$

### Sperm morphology

In each sample spermatozoa were evaluated and classified as previously reported in the bull [21], and the percentage of each abnormality, such as the total sperm abnormality, was calculated on at least 200 spermatozoa. The percentage of non-sperm cells was also estimated and recorded in samples. Epithelial cells, leukocytes and round cells were classified as non-sperm cells.

### Sperm kinematic evaluation

Objective sperm kinetic was measured with a computer assisted sperm analyzer (CASA) IVOS 12.3 (Hamilton-Thorne Bioscience, Beverly, MA, USA). An aliquot of each sample was rewarmed at 37 °C for 10 mins and analyzed in a Makler chamber (Sefi Medical Instruments, Haifa, Israel), as previously reported [22]. In this study, the following parameters were considered: total motility (TM, %), progressive motility (PM, %), average path velocity (VAP, μm/s), straight line velocity (VSL, μm/s), curvilinear velocity (VCL, μm/s), amplitude of lateral head displacement (ALH, μm), beat cross frequency (BCF, Hz), straightness (STR, as VSL/VAP, %), and linearity (LIN, as VSL/VCL, %). Spermatozoa with a VAP ≥ 80 μm/s and STR ≥ 75 % were considered progressive [17]. The CASA settings used in this study were those previously reported [23].

### Sperm membrane and acrosome integrity

The sperm membrane and acrosome integrity were evaluated simultaneously by flow cytometry. Samples (1 mL) were stained with 2.4 μmol of propidium iodide (PI) and 5 μg/mL of FITC-conjugated agglutinin derived from pisum sativum (FITC-PSA). After 10 mins of dark incubation at room temperature, each sample was analyzed by the flow cytometer EPICS XL (Beckman Coulter, San Jose, CA, USA). Acquisitions were conducted using the System II software (Beckman Coulter, USA). The samples were excited with a 20-mW argon ion 488-nm laser. The FITC-PSA fluorescence was collected in the FL1 sensor using a 530/28 nm band-pass, while the PI fluorescence was obtained using the FL3 sensor through a 660/20 nm long pass filter. Forward and side-scatter values were recorded on a linear scale and fluorescence values on a logarithmic scale. No compensation was used between the fluorescent channels. The flow cytometric analysis was performed at a flow rate of 6 to 24 μL/min, and the acquisitions were stopped at 30,000 events. This stain association resulted in four different subpopulations: sperm with membrane integrity and acrosome integrity showing no fluorescence (PI-/PSA-); sperm with membrane integrity and an acrosome reaction (PI-/PSA+); sperm with a damaged membrane and acrosome integrity (PI+/PSA-); and sperm with a damaged membrane and a reacted acrosome (PI+/PSA+).

### Mitochondrial membrane potential analysis

Mitochondrial membrane potential (MMP) of spermatozoa was evaluated using the fluorescent stain 5,5ʹ,6,6ʹ-tetrachloro-1,1ʹ,3,3ʹ-tetraethyl-benzimidazolylcarbocyanine chloride (JC-1). The lipophilic cationic fluorescent carbocyanine dye, JC-1, was used to differentially label mitochondria with high and low membrane potential [24]. The sperm suspension was adjusted to a density of 5 × 10^6^ sperm/mL and incubated for 45 mins at 37 °C in the dark with JC-1 probe (5 μmol). At the end of the incubation period, cells were washed in TALP and evaluated using the flow cytometer EPICS XL (Beckman Coulter) equipped with the System II software (Beckman Coulter) as previously reported [25]. The green fluorescent emissions of the monomeric form of JC-1 (mitochondria with low potential - lMMP) were collected by a 530 ± 15 - nm filter (FL 1), and the orange emission of the polymeric form of JC-1 (mitochondria with high membrane potential - hMMP) by a 585 ± 21 – nm filter (FL 2). The flow cytometric analysis was performed at a flow rate of 8 to 30 μL/min, and the acquisitions were stopped at 30,000 events. No compensation was used between the fluorescent channels.

### Statistical analysis

The data are presented as mean ± standard deviations (SD). Sperm characteristics for each treatment (SLC, DLC, CC and NC) at each evaluation point (before centrifugation, after centrifugation, after equilibration and after cryopreservation) in each bull group (normal and poor semen quality), were compared using a general linear model (GLM) based on the univariate ANOVA. The post-hoc analysis was performed using the Scheffè test. The bull was included as a random factor, in order to verify a possible effect on the results. In all cases, the differences were considered to be significant with P ≤ 0.05.

The statistical analyses were performed using the SPSS 17.0 software package (SPSS Inc. Chicago, IL, USA).

## Results

Raw semen characteristics from bulls with normal and poor semen quality involved in this study are summarized in Table 1. Sperm total and progressive motility, sperm velocities, and sperm morphology were significantly different in samples with normal and poor semen quality (*P* ≤ 0.05).

**Table 1:** Mean (±SD) of seminal parameters after collection in normal and poor quality ejaculates

Items	Normal semen quality	Poor semen quality
	Mean ± SD	Mean ± SD
Volume, mL	7.8 ± 2.5*	8.1 ± 1*
Concentration, × 10^6^ sperm/mL	1192 ± 203*	1048 ± 171*
Total sperm/ejaculate, × 10^6^ sperm/mL	10546 ± 3226*	9432 ± 1704*
Normal morphology, %	89.3 ± 3.7*	64.8 ± 11.6^§^
Non-sperm cells, %	0.02 ± 0.03*	2.9 ± 0.8^§^
TM, %	84 ± 7*	73 ± 4^§^
PM, %	73 ± 3*	59 ± 2^§^
VAP, μm/s	156.8 ± 20.4*	140.5 ± 11.7^§^
VSL, μm/s	131.8 ± 14.3*	119 ± 8.9^§^
VCL, μm/s	264.7 ± 40.8*	223.8 ± 17.6^§^
ALH, μm	9 ± 1.1*	8.7 ± 0.3*
BCF, Hz	44.6 ± 0.6*	42.9 ± 1.7*
STR, %	84 ± 2*	85 ± 2*
LIN, %	52 ± 3*	50 ± 1*
PI-, PSA-, %	80.4 ± 10.1*	73.5 ± 8.4*
PI-, PSA+, %	0.3 ± 0.1*	0.5 ± 0.2*
PI+, PSA-, %	16.7 ± 2.7*	21.9 ± 3.1*
PI+, PSA+, %	2.6 ± 0.3*	3.1 ± 0.5*
h-MMP, %	66.8 ± 5.4*	62.6 ± 6.9*

*TM* Total motility, *PM* progressive motility, *VAP* average path velocity, *VSL* straight line velocity, *VCL* curvilinear velocity, *ALH* amplitude of lateral head displacement, *BCF* beat cross frequency, *STR* straightness, *LIN* linearity, *PI-, PSA-* membrane integrity and acrosome integrity, *PI-, PSA+* membrane integrity and acrosome reaction, *PI+, PSA-* membrane damage and acrosome integrity, *PI+, PSA+* membrane damage and acrosome damage, *h-MMP* spermatozoa with high mitochondrial membrane potential

In the same row, values with different symbols in superscript (*/^§^) differ significantly (*P* ≤ 0.05)

Single layer centrifugation and DLC showed a reduced recovery rate in all samples. The mean recovery rate was 56 ± 11 % for SLC and 44 ± 14 % for DLC. No significant differences were found between the two methods. The CC showed a higher (*P* ≤ 0.05) recovery rate (84 ± 5 %).

In all samples from normal bulls, sperm morphology was similar before and after centrifugation. The type of abnormality was found to be dependent upon the individual bull (*P* ≤ 0.01), but no differences were found in the percentage of each type before and after centrifugation in different treatments in normal samples (data not shown). Similarly, in all N samples the proportion of non-sperm cells was low, with values between 0.06 and 0.1 %. The proportion of sperm with abnormal morphology decreased significantly in P bulls treated with SLC and DLC, reaching similar values compared with samples before centrifugation in N bulls. No differences were found in sperm abnormal morphology in untreated and CC samples before and after centrifugation in P bulls. In these bulls, the percentage of non-sperm cells significantly decreased (*P* ≤ 0.01) after SLC (from 2.4 ± 1.1 % to 0.2 ± 0.1 %) and DLC (from 3.1 ± 1.6 % to 0.08 ± 0.06 %), but not in samples conventionally centrifuged and non-centrifuged. In all samples the morphology of spermatozoa after equilibration and after cryopreservation were similar for the corresponding treatment.

After centrifugation, SLC and DLC samples showed an increased sperm kinetic parameters in P bulls. As showed in Table 2, total motility and progressive motility were higher in SLC and DLC samples from P bulls compared with the value recorded in poor quality NC and CC sperm (*P* ≤ 0.05). Sperm velocities after centrifugation were similar to those in untreated spermatozoa. Conventional centrifugation seemed to increase the ALH parameter in both normal and poor quality samples (*P* ≤ 0.05), while BCF, STR and LIN were similar among treatments.

**Table 2:** Mean (±SD) of sperm characteristics after different centrifugation treatments in normal (N) and poor (P) quality ejaculates

Items	SLC		DLC		CC		NC	
	N	P	N	P	N	P	N	P
	Mean ± SD	Mean ± SD	Mean ± SD	Mean ± SD	Mean ± SD	Mean ± SD	Mean ± SD	Mean ± SD
Normal morphology, %	90.4 ± 5.3*	91.2 ± 4.4*	89.6 ± 3.2*	91.3 ± 3.7*	85.9 ± 5.4*	63.7 ± 6.4^§^	87.9 ± 4.6*	66.2 ± 7.1^§^
Non-sperm cells, %	0.05 ± 0.04*	0.07 ± 0.04*	0.07 ± 0.05*	0.05 ± 0.03*	0.06 ± 0.03*	2.8 ± 1.1^§^	0.03 ± 0.02*	3.2 ± 1.3^§^
TM, %	91 ± 6*	91 ± 3*	91 ± 5*	92 ± 3*	85 ± 6^§^	86 ± 6^§^	84 ± 7^§^	83 ± 4^§^
PM, %	74 ± 10*	79 ± 4*	77 ± 3*	81 ± 1*	64 ± 6^§^	65 ± 5^§^	66 ± 3^§^	66 ± 2^§^
VAP, μm/s	153.2 ± 10.9*	153.5 ± 10.4*	150.1 ± 10*	147.6 ± 7.5*	152.7 ± 13.9*	153.3 ± 16.9*	156.8 ± 20.4*	140.5 ± 11.7^§^
VSL, μm/s	127.2 ± 9.4*	132.3 ± 11.7*	129.2 ± 8.6*	128.4 ± 7.7*	126.1 ± 9.9*	126.7 ± 16.5*	131.8 ± 14.3*	119 ± 8.9^§^
VCL, μm/s	256.6 ± 35.9*^§^	263.4 ± 18.5^§^	252.4 ± 25.8*	255.9 ± 14.1*	261.6 ± 25.7*	271.1 ± 19.3^§^	264.7 ± 40.8^§^	243.8 ± 17.6*
ALH, μm	8.9 ± 1.7*	8.7 ± 0.7*	8.6 ± 1.2*	8.8 ± 0.5*	9.3 ± 0.8^§^	9.6 ± 0.3^§^	8.8 ± 1.1*	8.7 ± 0.3*
BCF, Hz	44.3 ± 3.3*	45.2 ± 3*	45.2 ± 1.9*	44.7 ± 1.4*	43.8 ± 1.9*	42.7 ± 2.6*	44.6 ± 1.6*	42.9 ± 1.7*
STR, %	83 ± 3*	86 ± 2*	86 ± 4*	87 ± 2*	83 ± 2*	82 ± 3*	84 ± 2*	85 ± 2*
LIN, %	51 ± 3*	53 ± 2*	53 ± 3*	53 ± 2*	51 ± 2*	50 ± 2*	52 ± 1.5*	51 ± 2*
PI-, PSA-, %	86.9 ± 5.4*	85.1 ± 8.3*	83.7 ± 7.3*	84.5 ± 3*	75.5 ± 10.4^§^	72.3 ± 4.7^§^	82.4 ± 7.27*	71.3 ± 10.3^§^
PI-, PSA+, %	0.5 ± 0.2*	0.4 ± 0.2*	0.3 ± 0.2*	0.4 ± 0.3*	0.6 ± 0.2*	0.7 ± 0.3*	0.5 ± 0.3*	0.7 ± 0.4*
PI+, PSA-, %	10.3 ± 0.9*	12 ± 1.1*	13.8 ± 1.2*	12.6 ± 0.9*	20.8 ± 1.7^§^	24 ± 1.4^§^	14.9 ± 1.1*	24.9 ± 1.3^§^
PI+, PSA+, %	2.3 ± 0.4*	2.5 ± 0.3*	2.2 ± 0.3*	2.5 ± 0.2*	3.1 ± 0.4*	3 ± 0.3*	2.2 ± 0.3*	3.1 ± 0.4*
h-MMP, %	53.6 ± 4.2*	52.9 ± 3.8*	54.2 ± 4.6*	53.1 ± 4.1*	52.4 ± 3.7*	51.9 ± 3.2*	45.8 ± 3.2^§^	41.8 ± 6.4^§^

*TM* Total motility, *PM* progressive motility, *VAP* average path velocity, *VSL* straight line velocity, *VCL* curvilinear velocity, *ALH* amplitude of lateral head displacement, *BCF* beat cross frequency, *STR* straightness, *LIN* linearity, *PI-, PSA-*membrane integrity and acrosome integrity, *PI-, PSA+* membrane integrity and acrosome damage, *PI+, PSA-* membrane damage and acrosome integrity, *PI+, PSA+* membrane damage and acrosome damage, *h-MMP* spermatozoa with high mitochondrial membrane potential

In the same row, values with different symbols in superscript (*/^§^) differ significantly (*P* ≤ 0.05)

After equilibration for 3 h at 4 °C, the proportion of total and progressive motility was similar to the values recorded for the corresponding treatment after centrifugation, such as ALH, BCF, STR, and LIN. On the other hand, VAP, VSL, and VCL were significantly lower in untreated N and P samples (*P* ≤ 0.05), but were similar in all centrifuged samples (data not shown). After cryopreservation, a significant decrease in sperm total and progressive motility was recorded in all samples compared with the respective pre-freezing samples. However, NC normal samples showed similar values compared with normal and poor quality samples centrifuged with SLC and DLC, while poor quality non-centrifuged samples and CC normal and poor quality samples showed lower values for total and progressive motility (*P* ≤ 0.05). A significant decrease in ALH was found after cryopreservation (*P* ≤ 0.05), with higher values in CC samples (*P* ≤ 0.05). The other kinetic parameters were similar in all treatments (Table 3).

**Table 3:** Mean (±SD) of cryopreserved sperm characteristics different treatments in normal (N) and poor (P) quality ejaculates

Items	SLC		DLC		CC		NC	
	N	P	N	P	N	P	N	P
	Mean ± SD	Mean ± SD	Mean ± SD	Mean ± SD	Mean ± SD	Mean ± SD	Mean ± SD	Mean ± SD
TM, %	55.5 ± 5.2*	54.3 ± 7.2*	55.8 ± 8.6*	56.8 ± 5.6*	44.5 ± 15.5^§^	45 ± 11.5^§^	56.7 ± 6.3*	45.3 ± 9.1^§^
PM, %	42.7 ± 6.4*	43 ± 6.5*	41.5 ± 5.5*	43.8 ± 5.1*	25.8 ± 13.4^§^	28 ± 7.2^§^	42.1 ± 6.7*	34 ± 9.4*^§^
VAP, μm/s	124.8 ± 11.5*	122.3 ± 12.1*^§^	127.8 ± 16.1*	128.9 ± 18.4*	115.8 ± 17.2^§^	119 ± 10.9*^§^	127.4 ± 13.4*	116.7 ± 7.6^§^
VSL, μm/s	109.6 ± 15*	101.7 ± 10.1*	108.7 ± 15.7*	105.3 ± 18.7*	100.3 ± 18.1*	108.1 ± 10.3*	110 ± 14.5*	100.6 ± 9*
VCL, μm/s	193 ± 10*^§^	179.1 ± 17.4*	181.1 ± 27.5*	177.3 ± 35.7*	202.8 ± 19.4*^§^	225.5 ± 15.7^§^	212 ± 17.9*^§^	164.9 ± 6.2*
ALH, μm	7.4 ± 0.7*	6.9 ± 0.7*	7.7 ± 0.9*	7.1 ± 1.6*	8.3 ± 0.5^§^	8.6 ± 1.2^§^	7.7 ± 0.7*	7.5 ± 0.7*
BCF, Hz	38.6 ± 2.4*	37.1 ± 1.4*	33.8 ± 4.1*	33.4 ± 4.4*	36.8 ± 1.4*	35.7 ± 1.2*	38.7 ± 3.1*	35.8 ± 3.2*
STR, %	83.8 ± 4.4*	83.8 ± 2.9*	80.3 ± 1.9*	81.5 ± 2.5*	81.5 ± 4.4*	82 ± 1*	83.8 ± 2.8*	84.8 ± 3.2*
LIN, %	51.7 ± 5.9*	49.8 ± 3.8*	45.7 ± 2.7*	48 ± 2.2*	48 ± 5.7*	49 ± 1.7*	51.7 ± 3.4*	52.5 ± 3.7*
PI-, PSA-, %	51.3 ± 5.8*	50.8 ± 8.4*	52.5 ± 7.9*	51 ± 7.2*	42.6 ± 5^§^	38.9 ± 6.5^§^	54.3 ± 8.1*	39.4 ± 6.8^§^
PI-, PSA+, %	8.3 ± 2*	8.2 ± 1.2*	6.6 ± 1.4*	7.3 ± 1.8*	9.3 ± 2.4*	12.2 ± 4.5*	8 ± 2*	8.6 ± 1.1*
PI+, PSA-, %	26.1 ± 5.8*^§^	27.3 ± 5.9*^§^	22.6 ± 2.3*	24.5 ± 5.5*^§^	27.4 ± 6.1*^§^	27.1 ± 4.6*^§^	22.3 ± 3.3*	33.5 ± 6.8^§^
PI+, PSA+, %	14.3 ± 2.6*	13.7 ± 3.9*	18.3 ± 2.6*	17.2 ± 3.8*	20.7 ± 5.2*	21.9 ± 6.9*	15.4 ± 3.2*	18.5 ± 6.3*
h-MMP, %	34.1 ± 7.1*	35.7 ± 5.4*	38.6 ± 10.9*	31.6 ± 11.4*	33.7 ± 3.4*	30.9 ± 17.2*	35.9 ± 8.2*	32.6 ± 9.2*

*TM* Total motility, *PM* progressive motility, *VAP* average path velocity, *VSL* straight line velocity, *VCL* curvilinear velocity, *ALH* amplitude of lateral head displacement, *BCF* beat cross frequency, *STR* straightness, *LIN* linearity, *PI-, PSA-* membrane integrity and acrosome integrity, *PI-, PSA+* membrane integrity and acrosome reaction, *PI+, PSA-* membrane damage and acrosome integrity, PI+, *PSA+* membrane damage and acrosome damage, *h-MMP* spermatozoa with high mitochondrial membrane potential

In the same row, values with different symbols in superscript (*/^§^) differ significantly (*P* ≤ 0.05)

The membrane integrity of spermatozoa centrifuged with SLC and DLC in both N and P bulls were comparable with the membrane integrity of NC samples in N bulls. On the other hand, CC significantly decreased sperm membrane integrity in both N and P bulls, and the data recorded were similar to that in NC spermatozoa of P bulls (Table 2). A similar trend was found after equilibration. Surprisingly, sperm membrane integrity in samples after centrifugation or after equilibration was lower than sperm total motility in all treatments, suggesting that some spermatozoa with sub-lethal membrane damage could have flagellar beat. Cryopreservation dramatically impacted on sperm membrane integrity, since a significant decrease in sperm with membrane integrity was recorded in NC such as in SLC, DLC, and CC samples (Table 3). Sperm with reacted acrosomes were similar in all treatments and increased significantly only after cryopreservation (*P* ≤ 0.05).

A significant increase in sperm with high MMP was found in all centrifuged samples (SLC, DLC, and CC) compared with the NC samples soon after centrifugation, but the differences become not significant after cryopreservation. At this stage, spermatozoa of N bulls in NC samples showed similar values compared with SLC and DLC spermatozoa in both N and P bulls, and with CC spermatozoa in N bulls. On the other hand, a significantly lower proportion of spermatozoa with high MMP was found in NC and CC samples of P bulls (*P* ≤ 0.05) (Table 3).

## Discussion

In several cases the semen quality of high genetic bulls could be transiently or permanently decreased, resulting in the elimination of the batch from the cryopreservation procedure. This could result in a relevant economic loss for the artificial insemination centers. In a previous study gradient centrifugation was proposed as a method to select sperm subpopulation of normal size in a polymorphic bull [26]. In the present study the single and the double layer centrifugation procedure, by the use of iodixanol, significantly improved the quality of the poor quality bull samples.

In our study, sperm morphology significantly improved after SLC and DLC in poor quality samples. The abnormal spermatozoa were considered more buoyant compared to normal sperm [27], and it was apparent that the density of spermatozoa increased with maturation [28]. Although the latter parameter was not specifically considered in this study, the increase in the DNA integrity reported in several papers on spermatozoa collected in the pellet after SLC confirmed this hypothesis, in humans [29, 30] and animals [16, 31–33]. On the other hand, in samples of normal quality, sperm morphology after both SLC and DLC was similar compared to that of untreated or CC samples. The improvement of sperm morphology after gradient centrifugation is debated in literature, with studies where the sperm with normal morphology increases [32], and others in which this parameter was similar to the untreated samples [11, 16]. As in the latter cases, our results suggest that when the sperm morphological quality is high, the improvement is negligible. Single and double layer centrifugation seemed to remove also other cells and debris present in the ejaculate. In the samples with poor quality, epithelial cells, white blood cells, or cell debris, were reduced after centrifugation. Previous studies in horses showed that SLC or density gradient centrifugation [11] decreased the number of non-sperm cells in the pellet, and that this could reduce the formation of myeloperoxidase in the post-thaw samples [34]. This improvement is not reported in the cushioned centrifugation, in which both spermatozoa and cell debris are usually collected in the pellet [35, 36].

In poor quality samples, sperm kinetic characteristics significantly increased after SLC and DLC, with values similar to those reported in normal quality samples. Total and progressive motility increased after SLC and DLC compared with untreated and CC samples in all normal samples; this was evident also after equilibration. On the other hand, negligible effects were found on the other kinetic parameters in normal bulls, with the exception that ALH increased in CC samples, and the difference became significant after cryopreservation. Our findings were different to those reported in a previous study, in which total and progressive motility were found to be similar in samples untreated and centrifuged by single layer (SLC): sperm straightness, linearity, and beat cross frequency were significantly higher in SLC, whereas velocity of the average path, curvilinear velocity and amplitude of lateral head deviation all significantly decreased [37]. These differences in the kinematic aspect could be due to the treatment of the samples before analysis. In that study, bull semen was transported overnight at 4 °C before centrifugation and evaluation, and this could affect the results. Furthermore, a different device was used for the analysis of sperm kinematics, which could explain the difference in values reported in our trials. Our previous study has clarified that the technical characteristics of the computer assisted sperm analyzer could affect the results [23].

Sperm mitochondrial activity increased in poor quality samples after centrifugation, and this was maintained also during equilibration and cryopreservation. This could be related to the sperm kinetic parameters, since a similar trend was found for these characteristics. On the other hand, no differences were found in samples before and after centrifugation in normal bulls, in contrast with previous findings that showed a significant increase in mitochondrial membrane potential (from 56 to 83 %) after SLC [16].

The bull samples treated by SLC and DLC with iodixanol showed a similar sperm quality after cryopreservation compared with the untreated samples and the CC samples. This was in contrast with studies in the stallion [15, 38] and in the boar [39], in which the removal of seminal plasma improve the sperm resistance to the cryoinjury. Furthermore, Heutelbeck et al. [12] hypothesized that the higher sperm membrane integrity after cryopreservation in samples processed using a two-layer iodixanol density centrifugation was due to the cryoprotective effect of the iodixanol [40]. This hypothesis was unconfirmed by our data, since the both samples treated with iodixanol (SLC and DLC) showed similar parameters after thawing compared with untreated samples. A possible explanation is that the concentration of the iodixanol could be too low to give a cryoprotective effect.

In samples centrifuged using the conventional procedure (CC samples) total and progressive motility decreased significantly in both normal and poor quality samples after cryopreservation, but this effect was not present soon after centrifugation or equilibration and apparently did not involve the other kinetic properties. Conventional centrifugation results in a sperm pellet formation at the bottom of the centrifugation tube, and this is believed to have a negative effect on spermatozoa [13–15]. These results suggested that latent damage, not visible during liquid storage, could be present as a consequence of the centrifugation stress, but this effect was not elicited during liquid storage, and only after cryopreservation. However, specific trials to verify this hypothesis should be performed.

One of the problems related to the applicability of density centrifugation was the loss of spermatozoa. In the present study the recovery rate recorded in samples of normal quality centrifuged using SLC (56 %) and DLC (44 %) was significantly lower compared with the CC method (84 %). The data reported here were comparable with those reported using a DLC in the stallion [11], in which the iodixanol density gradient centrifugation allowed a recovery rate of 33 %, while the ordinary centrifugation reached a 74 % recovery rate. Several studies in different species showed that SLC, by the use of silane-coated silica colloid, resulted in a significant loss of spermatozoa, with a yield rate ranging from 30 to 65 % [32] in horses, and a 50 % in bulls [9]. The higher recovery rate reported in this study compared with data reported in the SLC using silane-coated silica colloid could be the result of the variations in g-force used for the centrifugation. The centrifugation speed could be considered one of the most important factors affecting sperm recovery during CC [15], and this could be applied also to SLC. The density centrifugation was in all cases related to a reduced amount of spermatozoa recovered after centrifugation. Thus, it was concluded that this procedure may be inapplicable in practice when semen of normal quality should be handled. However, it could be considered when the sperm characteristics are of greater importance than the total amount of spermatozoa recovered after density centrifugation [12]. This is the case of our study, in which sperm of low quality were treated by SLC and DLC. In both treatments, sperm characteristics significantly improved.

The increase in recovery rate could be obtained by the increase in the centrifugation force. Cushioned centrifugation was proposed to increase the recovery rate of spermatozoa by higher gravitational force avoiding harmful effects on semen [41, 42]. During cushioned centrifugation, the use of a dense cushion, mainly iodixanol, in the bottom of the centrifugation tube was used to avoid the excessive compaction of the sperm pellet during high speed centrifugation [43]. The data reported in literature showed that a centrifugation speed exceeding 1,800 × g could be deleterious for equine sperm motility [44], while centrifugation at 600 × g [45], 900 × g [44], and 1,000 × g [43] had a negligible effect on equine sperm characteristics. However, the data reported in the present study showed that the presence of a cushion (DLC) did not improve the seminal quality either after centrifugation, or after equilibration or cryopreservation, compared with SLC.

## Conclusions

In this study the effects of single layer centrifugation and double layer centrifugation on bull sperm characteristics after cryopreservation in normal and poor quality ejaculates were studied. In bulls with normal semen characteristics, no significant effects were found in samples before and after SLC and DLC compared with untreated samples. Single layer centrifugation and DLC seemed to be useful tools to improve sperm characteristics of poor quality semen in bulls of high genetic value, where the sperm characteristics after both these procedures were comparable to those of normal samples. Thus, these procedures could be applicable in practice when the quality of the spermatozoa recovered is of greater importance than the total amount of spermatozoa.

## Abbreviations

AI: artificial insemination
ALH: amplitude of lateral head displacement
BCF: beat cross frequency
CASA: computer assisted sperm analyzer
CC: conventional centrifugation
DLC: double layer centrifugation
FITC-PSA: FITC-conjugated agglutinin derived from pisum sativum
JC-1: 5,5ʹ,6,6ʹ-tetrachloro-1,1ʹ,3,3ʹ-tetraethyl-benzimidazolylcarbocyanine chloride
LIN: linearity
MMP: Mitochondrial membrane potential
N: normal quality semen
NC: non-centrifuged
P: poor quality semen
PI: propidium iodide
PM: progressive motility
ROS: reactive oxygen species
SLC: single layer centrifugation
STR: straightness
TALP: tyrode’s medium
TM: total motility
VAP: average path velocity
VCL: curvilinear velocity
VSL: straight line velocity
